# Flexible identification procedure for thermodynamic constitutive models for magnetostrictive materials

**DOI:** 10.1098/rspa.2018.0280

**Published:** 2019-03-27

**Authors:** Paavo Rasilo, Deepak Singh, Juha Jeronen, Ugur Aydin, Floran Martin, Anouar Belahcen, Laurent Daniel, Reijo Kouhia

**Affiliations:** 1 Tampere University, Electrical Engineering, PO Box 692, 33014 Tampere University, Finland; 2 Department of Electrical Engineering and Automation, Aalto University, PO Box 15500, 00076 Aalto, Finland; 3 Tampere University Civil Engineering, PO Box 600, 33014 Tampere University, Finland; 4 GeePs | Group of electrical engineering – Paris, UMR CNRS 8507, CentraleSupélec, Univ. Paris-Sud, Université Paris-Saclay, Sorbonne Université, 3 rue Joliot-Curie, Plateau de Moulon, Gif-sur-Yvette 91192, France

**Keywords:** constitutive laws, magnetic materials, magnetoelasticity, magnetostriction, splines

## Abstract

We present a novel approach for identifying a multiaxial thermodynamic magneto-mechanical constitutive law by direct bi- or trivariate spline interpolation from available magnetization and magnetostriction data. Reference data are first produced with a multiscale model in the case of a magnetic field and uniaxial and shear stresses. The thermodynamic model fits well to the results of the multiscale model, after which the models are compared under complex multiaxial loadings. A surprisingly good agreement between the two models is found, but some differences in the magnetostrictive behaviour are also pointed out. Finally, the model is fitted to measurement results from an electrical steel sheet. The spline-based constitutive law overcomes several drawbacks of analytical approaches used earlier. The presented models and measurement results are openly available.

## 1. Introduction

Modelling tools for coupled magneto-mechanical effects in magnetic materials are needed for analysing losses and vibrations in electrical machines, as well as magnetostrictive devices and actuators. The problem is rather complex due to its three-dimensional and multiaxial nature, since many different combinations and orientations of magnetic fields and mechanical stresses can occur in real applications. In electrical machines, mechanical stresses typically cause adverse effects by increasing the losses [1–3]. On the other hand, the same effects allow converting mechanical energy into electrical energy for energy-harvesting purposes [4–6]. The coupled magneto-mechanical effects have been studied experimentally [7–10], while the modelling in the field has evolved from purely uniaxial studies [11] to equivalent stress or strain approaches for multiaxial loadings [12–15], and further towards genuinely multiaxial models [16–19].

Thermodynamic approaches provide one possible way of deriving coupled constitutive laws. Couplings between magnetic, electric and thermal fields [20–22] as well as elastic and plastic deformations [23–26] have been considered through thermodynamic frameworks. The applications have included both isotropic [20,22] and anisotropic [26] solids as well as granular materials [27–31]. In [32], an extensive review of earlier magneto-elastic constitutive models is given, and implicit constitutive equations based on 21 scalar invariants are derived for bodies under large deformations. These equations are then simplified for small fields and applied for solving magneto-elastic boundary value problems for slabs and an annulus. However, systematic procedures for identification of such models based on experimental data have not been reported in details so far. It is particularly mentioned in [32] that the given equations are ‘too general to be used to correlate with experimental data as there are so many material functions that depend on numerous invariants.' In this paper, we focus on identification of thermodynamic constitutive models based on the theory of invariants.

In our earlier works, we have developed a thermodynamic approach for deriving coupled magneto-mechanical constitutive equations for ferromagnetic materials [33]. The approach has been based on expressing a Helmholtz free energy per unit volume *ψ*(***B***, ***ε***) as a function of the magnetic flux density vector ***B*** and total small strain tensor ***ε***, i.e. the symmetric part of the displacement gradient. The magnetization and stress have then been derived by the Coleman–Noll procedure from the dissipation inequality, and are given by

1.1 $$M=-{\left(\frac{\partial \psi }{\partial B}\right)}^{\text{T}}\;\text{and}\;\sigma =\frac{\partial \psi }{\partial \varepsilon }.$$

The field strength then becomes

1.2 $$H=\frac{1}{\mu_{0}}B-M,$$

where *μ*_0_ is the permeability of free space. By assuming an isotropic material, the dependency of the Helmholtz free energy density on ***B*** and ***ε*** reduces to the following six invariants

1.3 $$\begin{array}{rl} I_{1} & =\text{tr }\varepsilon ,\;I_{2}=\text{tr }\varepsilon^{2},\;I_{3}=\text{tr }\varepsilon^{3},\\ I_{4} & =B\cdot B,\;I_{5}=B\cdot eB,\;I_{6}=B\cdot e^{2}B, \end{array}$$

where $e=\varepsilon -\frac{1}{3}(\text{tr }\varepsilon )I$ is the deviatoric strain, ***I*** being the second-order identity tensor and tr denotes the trace of a tensor. Equation (1.1) thus becomes

1.4 $$M=-{\left(\sum_{i=1}^{6}\frac{\partial \psi }{\partial I_{i}}\frac{\partial I_{i}}{\partial B}\right)}^{\text{T}}\;\text{and}\;\sigma =\sum_{i=1}^{6}\frac{\partial \psi }{\partial I_{i}}\frac{\partial I_{i}}{\partial \varepsilon },$$

which are uniquely defined when *ψ*(*I*_1_, *I*_2_, *I*_3_, *I*_4_, *I*_5_, *I*_6_) is known.

So far, we have mainly used analytical expressions for *ψ*(*I*_1_, *I*_2_, *I*_3_, *I*_4_, *I*_5_, *I*_6_). The exact form of the function has varied (see different variations in [33–38]), but has been more or less similar to

1.5 $$\psi =\frac{1}{2}\lambda I_{1}^{2}+\mu I_{2}+\sum_{i=1}^{n_{\alpha }}\alpha_{i}I_{4}^{i}+\sum_{i=1}^{n_{\beta }}\beta_{i}I_{5}^{i}+\sum_{i=1}^{n_{\gamma }}\gamma_{i}I_{6}^{i},$$

in which λ and *μ* are the Lamé parameters yielding linear Hooke's Law (appendix A), and the nonlinear magneto-elastic part is described by the last three polynomials with *α_i_*, *β_i_* and *γ_i_* as fitting parameters. Invariant *I*_3_ has usually been neglected in order to reproduce linear behaviour under purely mechanical loading. Although these analytical expressions are relatively easy to implement, they have sometimes failed to properly fit to measured magnetization and magnetostriction curves. More complicated analytical expressions for the magneto-elastic part might improve the fitting, but finding a suitable expression is rather time-consuming and does not bring any added physical insight into the phenomena. In addition, if the state variables are to be changed, a different analytical expression might need to be used. Analytical expressions for the energy functions were also used in [21] and [22].

In this paper, we propose a new approach for overcoming the disadvantages of analytical energy density expressions. The idea is to express the energy density as a spline of the invariants, identifying the free energy density function as a direct least-squares spline fit to measured magnetization and magnetostriction curves. The main advantage of the proposed approach compared to [33–38] is that no *a priori* assumptions on how to analytically express the free energy density need to be made. This provides more flexibility when fitting the model against measurements, and also allows keeping the model fitting procedure unchanged, even if the input variables need to be changed. Using ***B*** as the magnetic input variable is convenient if the constitutive law is to be used in combination with a finite-element (FE) formulation based on the magnetic vector potential, while ***H*** is more suitable for magnetic scalar potential formulations. Using ***ε*** as the mechanical input variable is convenient in displacement-based FE formulations, while using ***σ*** is often straightforward in simple statically determined structures.

In the following, we first describe our measurement set-up and demonstrate the problems related to fitting the analytical energy density expression (1.5) against measurements from an electrical steel sheet. The new spline-based approach overcoming the problems of the analytical approach is then described. The splines are fitted to simulation results produced by the simplified multiscale (SMS) model of [17], and the behaviour of the models under complex multiaxial loadings is compared. Finally, it is demonstrated that the proposed approach provides a significantly better fit against measurements than the analytical expression (1.5).

## 2. Methods

### (a) Measurement set-up and problems with the analytical models

A custom-designed stressing mechanism was built offering the possibility of in-plane uniaxial stressing of a modified single sheet tester (SST) sample. The mechanism was designed to exert both compressive and tensile stress up to 100 MPa for a 25 × 0.5 mm cross-sectioned SST sample. A manual two-way locking screw system in series with a helical compression spring was used to exert the required force. Use of spring was especially considered to better control the stress levels, thus a force resolution of 1 N was achieved with this device. In addition to the stressing mechanism, designing the SST sample is essential as well. In order to achieve a uniform stress at the measurement zone, i.e. centre of the SST sample, proper propagation of force from the clamped zone to the centre of the sample is required. Thus the shape of the SST sample is chosen according to tensile testing standards.

**Figure 1.**
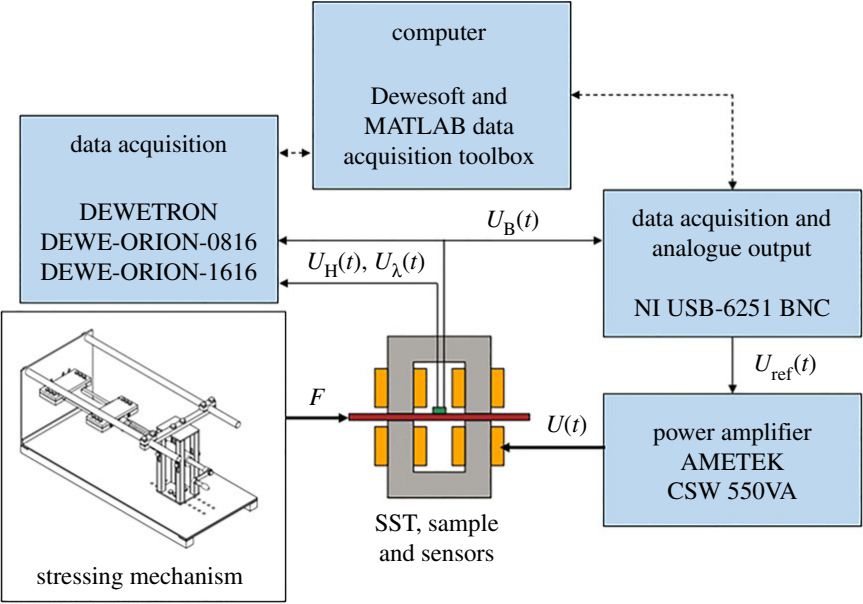
Schematic of the uniaxial measurement set-up. The stressing mechanism applies a static force *F* into the sample. The SST is excited by a voltage waveform *U*(*t*) and the voltage waveforms from the field strength sensor (*U*_H_(*t*)), strain gauge (*U_λ_*(*t*)) and search coil (*U*_B_(*t*)) are measured. An input voltage waveform *U*_ref_(*t*) is iteratively searched such that sinusoidal *U*_B_(*t*) is obtained. (Online version in colour.)

For the magnetic measurements, tunnelling magneto-resistance (TMR) sensors of dimension 6 × 5 × 1.5 mm arranged in a 2 × 2 grid on a printed circuit board of 20 × 20 mm were used to measure the surface magnetic field strength. When placed on the SST sample, the sensing element of the TMR sensor chips was effectively at 0.4–0.5 mm above the sample surface. Similarly, a search coil wound around the SST sample was used to measure the average magnetic flux density. At the low measurement frequency of 6 Hz, the demagnetization field by the eddy currents is small and the surface field strength and average flux density give a sufficient approximation of the static constitutive behaviour. Furthermore, a non-inductive three element strain gauge rosette of 60° delta arrangement (H-series rosette from Micro-Measurements), glued on the surface of the sample (with the insulation coating removed) was used to measure the magnetostriction. The individual element of the rosette has the gauge length and resistance of 3.18 mm and 700 Ω, respectively. Figure 1 shows a schematic diagram of the measurement set-up.

A programmable power source (AMETEK CSW 5550VA) and a data acquisition system (DAQ-NI USB-6251 BNC) with analogue output were used in conjunction with a PC to control the magnitude and the waveform of the supply voltage so as to produce a sinusoidal induction in the SST sample. The feedback control of the supply voltage was programmed using the Matlab/DAQ toolbox. Low-noise/high-gain signal amplifiers were also used to amplify the signal obtained from the search coils. In addition to that, a high sampling rate DAQ system (DEWETRON) controlled by PC/DEWEsoft was used to retrieve the measured signals for the field strength and the flux density. The sample was magnetized using two vertical cores and the excitation coils with a total of 1000 turns. Within the limitation of the power amplifier's operation, the total turns of the excitation coils were sufficient for a wide range of induction amplitudes and frequencies.

**Figure 2.**
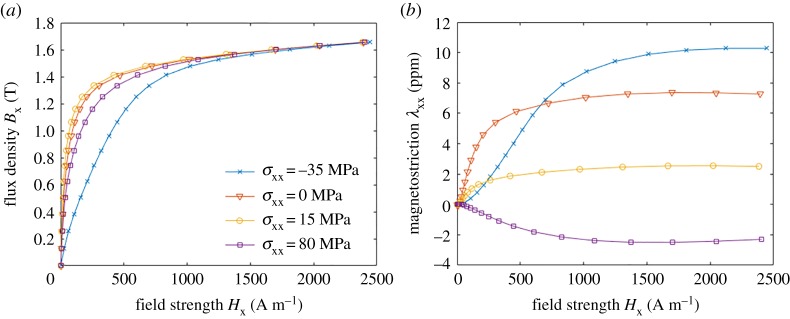
Measurement results of (*a*) magnetic flux density and (*b*) magnetostriction as functions of the magnetic field strength and uniaxial stress parallel to the field in M400-50A electrical steel. (Online version in colour.)

Figure 2 shows measurement results for magnetization curves and magnetostriction curves under different compressive (−) and tensile (+) stresses in M400–50A electrical steel. The single-valued curves have been obtained by averaging the hysteresis loops in order to identify anhysteretic material models. Although ferromagnetic hysteresis is a major source of losses in electromagnetic devices, anhysteretic material properties are usually sufficient to estimate the magnetic field distribution in devices with air gaps, and hysteresis losses can be calculated *a posteriori* with a reasonable accuracy [39,40]. Thus, only anhysteretic constitutive laws are considered in this paper.

**Figure 3.**
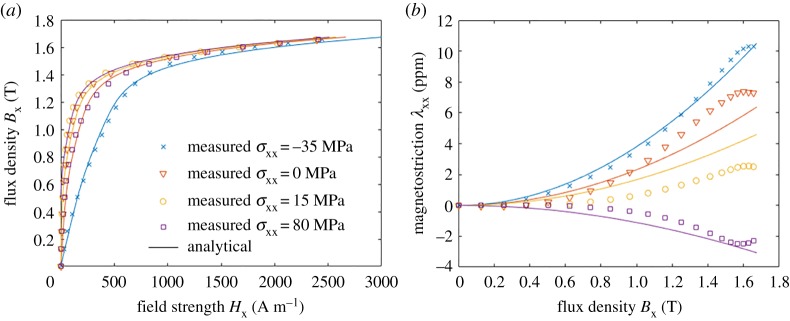
Fitting of the analytical energy-density expression *ψ*(***B***, ***ε***) given in (1.5) to the measurement results shown in figure 2: (*a*) field strength *H*_x_ and (*b*) magnetostriction λ_xx_. (Online version in colour.)

The problem related to the analytical energy density expressions is demonstrated by setting *n_α_* = 5, *n*_β_ = 1 and *n_γ_* = 1 in (1.5) and fitting *α_i_*, *β_i_* and *γ_i_* against the measurement results. In the fitting, the magnetization and magnetostriction curves derived from (1.5) at four different stress values were compared against the measured curves. The curves were normalized by their maximum values to give equal weights to the magnetization and magnetostriction curves. Since the stress is uniaxial in the measurements, the components of ***ε*** were first iterated separately for each value of *B*_x_ such that the desired stress was obtained. This makes the problem nonlinear with respect to the parameters and requires using a nonlinear minimization algorithm. The fitting was attempted using both nonlinear least-squares minimization and a genetic algorithm (Matlab functions **lsqcurvefit** and **ga**). Figure 3 shows the best results obtained from the fitting. Although the magnetization curves correspond relatively well to the measurements, the magnetostriction curves do not. This is caused by the limitations in the analytical expression (1.5), which fails to correctly describe the physical behaviour of the material. Derivation of a better expression would require a time-consuming trial and error process and would probably lead to complex analytical formulae. In addition, finding suitable initial values for the nonlinear fitting problem may be difficult. In the following, we propose a new identification approach based on linear least-squares spline fitting which does not require analytically expressing the energy density.

### (b) Fitting of the thermodynamic model in terms of *H* and *σ*

From now on, we focus mainly on the magneto-elastic part of the free energy density *ψ* discussed in §1, and denote this by *ϕ*. In addition, *ψ* and *ϕ* are referred to simply as *energy densities* (instead of Helmholtz energy density), since their physical meaning varies depending on the choice of state variables. We start by deriving the thermodynamic model by choosing the magnetic field strength vector ***H*** and the stress tensor ***σ*** as the state variables. This choice is preferred, since it will allow straightforward comparison to the SMS model of [17], which uses the same state variables. In this case, the relevant invariants become *I*_4_ = ***H ***· ***H***, *I*_5_ = ***H ***· ***sH*** and *I*_6_ = ***H ***· ***s***^2^***H***, where $s=\sigma -\frac{1}{3}(\text{tr }\sigma )I$ is the deviatoric stress. The magnetic flux density ***B*** and magnetostriction tensor **λ** can then be obtained as

2.1 $$B={\left(\frac{\partial \phi }{\partial H}\right)}^{\text{T}}\;\text{and}\;\lambda =\frac{\partial \phi }{\partial \sigma }.$$

It is noted that if the magnetic polarization *μ*_0_***M*** is preferred as the output variable instead of ***B***, the energy can be changed to $\phi \to \phi -\frac{1}{2}\mu_{0}I_{4}$ without affecting the magnetostriction. However, we prefer to write the constitutive models directly in terms of ***B*** and ***H*** since these are the variables of interest when numerically solving Maxwell equations.

The measurement set-up described in §2a allows characterizing the magneto-mechanical constitutive behaviour in the form of ***B***(***H***, ***σ***) and **λ**(***H***, ***σ***), where ***σ*** is uniaxial and parallel to ***B*** and ***H*** (figure 2). Devices for biaxial stressing [13] and shear stressing [10] have also been presented. In the following derivations, it will become apparent that consideration of uniaxial and shear stresses is sufficient to identify the complete energy density. Let us thus assume for now that ***H*** = [*H*_x_ 0 0]^T^, ***B*** = [*B*_x_ *B*_y_ 0]^T^, ***σ*** = [$\sigma_{\mathrm{xx}}$ 0 0 0 0 $\sigma_{\mathrm{xy}}$]^T^ and **λ** = [λ_xx_ λ_yy_ λ_zz_ 0 0 λ_xy_]^T^ (in Voigt notation ***σ*** = [$\sigma_{\mathrm{xx}}$ $\sigma_{\mathrm{yy}}$ $\sigma_{\mathrm{zz}}$ $\sigma_{\mathrm{yz}}$ $\sigma_{\mathrm{zx}}$ $\sigma_{\mathrm{xy}}$]), so that the invariants become

2.2 $$I_{4}=H_{\text{x}}^{\text{2}},\;I_{5}=\frac{2}{3}\sigma_{\text{xx}}H_{\text{x}}^{\text{2}}\;\text{and}\;I_{6}=\left(\frac{4}{9}\sigma_{\text{xx}}^{\text{2}}+\sigma_{\text{xy}}^{\text{2}}\right)H_{\text{x}}^{\text{2}}.$$

Since the material is assumed to be isotropic, only stress-induced magnetic anisotropy is present, and *B*_y_ can be non-zero only if $\sigma_{\mathrm{xy}}$ ≠ 0. Under uniaxial stress, $\sigma_{\mathrm{xy}}$ = 0, and only two of the invariants are independent. This means that we can identify the energy density only as a bivariate function, which is denoted *ϕ*^(2)^(*I*_4_, *I*_5_). However, if measurement data were available also for $\sigma_{\mathrm{xy}}$ ≠ 0, we could identify trivariate *ϕ*^(3)^(*I*_4_, *I*_5_, *I*_6_). It is emphasized, that with the given magnetic field in the *x*-direction, *I*_6_ is independent of *I*_4_ and *I*_5_ only if at least one of the shear stress components $\sigma_{\mathrm{xy}}$ or $\sigma_{\mathrm{zx}}$ is non-zero.

In this paper, we aim for a general identification approach, which does not require making assumptions on the form of functions *ϕ*^(2)^(*I*_4_, *I*_5_) or *ϕ*^(3)^(*I*_4_, *I*_5_, *I*_6_). Thus, instead of analytical expressions for the functions, we express them as bi- and trivariate B-splines of the input variables. The splines are then fitted directly on the measured ***B*** and **λ**. However, the difficulty is that the values of *H*_x_, $\sigma_{\mathrm{xx}}$ and $\sigma_{\mathrm{xy}}$, on which ***B*** and **λ** are measured, are often chosen so that they lie on a regular (but not necessarily uniform) grid. This means that the invariants *I*_4_, *I*_5_ and *I*_6_ will be irregularly distributed, which makes it difficult to construct the splines. To avoid this problem, we define auxiliary variables

2.3 $$u=\sqrt{I_{4}},\;v=\frac{3}{2}\frac{I_{5}}{I_{4}}\;\text{and}\;w=\frac{1}{I_{4}}\sqrt{I_{6}I_{4}-I_{5}^{2}},$$

which under the aforementioned ***H*** and ***σ*** become *u* = *H*_x_, *v* = $\sigma_{\mathrm{xx}}$ and *w* = $\sigma_{\mathrm{xy}}$, which are regularly distributed. In addition, with these variables, we obtain

2.4 $$\frac{\partial \phi }{\partial u}=B_{\text{x}},\;\frac{\partial \phi }{\partial v}=\lambda_{\text{xx}}\;\text{and}\;\frac{\partial \phi }{\partial w}=2\lambda_{\text{xy}}.$$

We can thus identify the splines *ϕ*^(2)^(*u*, *v*) and *ϕ*^(3)^(*u*, *v*, *w*) directly from the measured flux density and magnetostriction. Note the factor 2 in the last term of (2.4), which results from the fact that the symmetry assumption λ_yx_ = λ_xy_ is done before differentiating the energy. It is emphasized, that after the splines are identified, the auxiliary variables (2.3) and the splines are well defined for arbitrary three-dimensional field and stress configurations.

To formulate the spline fitting problem, let us assume that the regular grid formed by *H*_x_, $\sigma_{\mathrm{xx}}$ and $\sigma_{\mathrm{xy}}$ consists of *N*_u_, *N*_v_ and *N*_w_ values of each variable, respectively. The energy densities are expressed as bi- and trivariate B-splines of *u*, *v* and *w*. The spline coefficients are denoted *c_lm_* and *c_lmn_* for the bi- and trivariate cases, respectively, where *l* = 1, … ,*M*_u_, *m* = 1, … ,*M*_v_ and *n* = 1, … ,*M*_w_. To avoid overfitting, we typically want to have at most as many coefficients as measurement points: *M*_u_ ≤ *N*_u_, *M*_v_ ≤ *N*_v_ and *M*_w_ ≤ *N*_w_. The bi- and trivariant energy densities in the grid points thus become

2.5 $$\phi_{ij}^{\left(2\right)}=A_{il}^{\text{u}}A_{jm}^{\text{v}}c_{lm}\;\text{and}\;\phi_{ijk}^{\left(3\right)}=A_{il}^{\text{u}}A_{jm}^{\text{v}}A_{kn}^{\text{w}}c_{lmn},$$

where *i* = 1, … ,*N*_u_, *j* = 1, … ,*N*_v_ and *k* = 1, … ,*N*_w_ denote the indices of the measurement points, and the *A*-terms denote elements of collocation matrices ***A***^u^, ***A***^v^ and ***A***^w^ which map the B-spline coefficients into the energy densities in the measurement points, and which depend only on the chosen grid and the order of the B-spline. In the notation, the repeated subindices are summed over. If the energy densities in the measurement grid were measured directly, the spline coefficients could be directly solved from (2.5). However, since only the partial derivatives (2.4) are measured, we end up with

2.6 $$\begin{array}{rl} & \left\{ \begin{array}{l} B_{\text{x},ij}=D_{io}^{\text{u}}A_{jm}^{\text{v}}c_{om}\\ \lambda_{\text{xx, }ij}=A_{il}^{\text{u}}D_{jo}^{\text{v}}c_{lo} \end{array}\right.\\ \;\text{and}\; & \left\{ \begin{array}{l} B_{\text{x},ijk}=D_{io}^{\text{u}}A_{jm}^{\text{v}}A_{kn}^{\text{w}}c_{omn}\\ \lambda_{\text{xx},ijk}=A_{il}^{\text{u}}D_{jo}^{\text{v}}A_{kn}^{\text{w}}c_{lon}\\ \lambda_{\text{xy},ijk}=\frac{1}{2}A_{il}^{\text{u}}A_{jm}^{\text{v}}D_{ko}^{\text{w}}c_{lmo} \end{array}\right., \end{array}$$

where the *D*-terms are elements of collocation matrices ***D***^u^, ***D***^v^ and ***D***^w^, which map the spline coefficients into the measured partial derivatives. The problem is overdetermined. In the bivariate case, we have 2*N*_u_*N*_v_ equations for *M*_u_*M*_v_ variables, and in the trivariate case 3*N*_u_*N*_v_*N*_w_ equations for *M*_u_*M*_v_*M*_w_ variables. Details of the solution will be discussed in §2d. The order of the B-splines can be chosen freely, but it should be greater or equal to 3 to make the second derivatives continuous, which is beneficial when solving nonlinear field problems using Newton–Raphson iteration. Outside of the range of measured data, the splines are extrapolated as quadratic functions, meaning linear extrapolation for the partial derivatives. This is a reasonable assumption if identification data is available close up to saturation, since both the ***B***(***H***) and ***ε***(***σ***) relationships tend to become linear above saturation.

### (c) Fitting of the thermodynamic model in terms of *B* and *ε*

As mentioned in the previous section, measurements are typically available under known stress. Formulation of the model using the strain as the state variable thus needs some more considerations. Let us assume now that instead of ***H*** and ***σ***, the state variables are ***B*** and ***ε***, where ***ε*** = ***C***^–1^***σ*** + **λ** is the total strain (***C*** being the mechanical material stiffness matrix related to Hooke's Law and defined by Young's modulus *E* and Poisson's ratio *ν*). This choice of state variables is convenient, if the constitutive law is to be implemented in an FE formulation using the magnetic vector potential and mechanical displacement. The relevant invariants now become *I*_1_ = tr ***ε***, *I*_2_ = tr ***ε***^2^, *I*_4_ = ***B***·***B***, *I*_5_ = ***B ***· ***eB*** and *I*_6_ = ***B ***· ***e***^2^***B***, where $e=\varepsilon -\frac{1}{3}(\text{tr }\varepsilon )I$. The total energy density function becomes

2.7 $$\psi (I_{1},I_{2},I_{4},I_{5},I_{6})=\frac{1}{2}\lambda I_{1}^{2}+\mu I_{2}+\phi (I_{4},I_{5},I_{6}),$$

where the first two terms again give the linear isotropic elasticity equations and *ϕ*(*I*_4_, *I*_5_, *I*_6_) is to be expressed as a spline similarly to the previous section. The field strength and stress are obtained as

2.8 $$H={\left(\frac{\partial \psi }{\partial B}\right)}^{\text{T}}\;\text{and}\;\sigma =\frac{\partial \psi }{\partial \varepsilon },$$

which is consistent to (1.1) and (1.2) if a substitution $\psi \to \psi -\frac{1}{2}\mu_{0}^{-1}I_{4}$ is made to the energy density in (1.1).

Let us assume that measurements are still available under known stress in the form of ***H***(***B***, ***σ***) and **λ**(***B***, ***σ***), now with ***B*** = [*B*_x_ 0 0]^T^, ***H*** = [*H*_x_ *H*_y_ 0]^T^, ***σ*** = [$\sigma_{\mathrm{xx}}$ 0 0 0 0 $\sigma_{\mathrm{xy}}$]^T^ and **λ** = [λ_xx_ λ_yy_ λ_zz_ 0 0 λ_xy_]^T^ for regularly distributed *B*_x_, $\sigma_{\mathrm{xx}}$ and $\sigma_{\mathrm{xy}}$. The invariants now become

2.9 $$I_{4}=B_{\text{x}}^{\text{2}},\;I_{5}=e_{\text{xx}}B_{\text{x}}^{\text{2}}\;\text{and}\;I_{6}=(e_{\text{xx}}^{\text{2}}+\varepsilon_{\text{xy}}^{\text{2}})B_{\text{x}}^{\text{2}},$$

where *e*_xx_ is the xx-component of the deviatoric strain. Magnetostriction is assumed to be isochoric so that

2.10 $$\text{tr }\lambda =\lambda_{\text{xx}}+\lambda_{\text{yy}}+\lambda_{\text{zz}}=0.$$

The diagonal strain components are

2.11 $$\varepsilon_{\text{xx}}=\frac{\sigma_{\text{xx}}}{E}+\lambda_{\text{xx}},\;\varepsilon_{\text{yy}}=-\frac{\nu }{E}\sigma_{\text{xx}}+\lambda_{\text{yy}}\;\text{and}\;\varepsilon_{\text{zz}}=-\frac{\nu }{E}\sigma_{\text{xx}}+\lambda_{\text{zz}},$$

and the xx-component of the deviatoric strain is thus

2.12 $$e_{\text{xx}}=\frac{2}{3}\varepsilon_{\text{xx}}-\frac{1}{3}(\varepsilon_{\text{yy}}+\varepsilon_{\text{zz}})=\frac{2}{3}\frac{\nu +1}{E}\sigma_{\text{xx}}+\frac{2}{3}\lambda_{\text{xx}}-\frac{1}{3}(\lambda_{\text{yy}}+\lambda_{\text{zz}})=\frac{2}{3}\frac{\nu +1}{E}\sigma_{\text{xx}}+\lambda_{\text{xx}}.$$

In addition, the shear component of the strain can be written as

2.13 $$\varepsilon_{\text{xy}}=\frac{1+\nu }{E}\sigma_{\text{xy}}+\lambda_{\text{xy}},$$

and the invariants *I*_5_ and *I*_6_ thus become

2.14 $$I_{5}=\left(\frac{2}{3}\frac{1+\nu }{E}\sigma_{xx}+\lambda_{\text{xx}}\right)B_{\text{x}}^{\text{2}}\;\text{and}\;I_{6}=\left({\left(\frac{2}{3}\frac{1+\nu }{E}\sigma_{xx}+\lambda_{\text{xx}}\right)}^{2}+{\left(\frac{1+\nu }{E}\sigma_{\text{xy}}+\lambda_{\text{xy}}\right)}^{2}\right)B_{\text{x}}^{\text{2}}.$$

Using the same definitions as in (2.3) for the auxiliary variables, they become

2.15 $$u=B_{\text{x}}^{\text{2}},\;v^{\prime }=\frac{1+\nu }{E}\sigma_{xx}+\frac{3}{2}\lambda_{\text{xx}}\;\text{and}\;w^{\prime }=\frac{1+\nu }{E}\sigma_{\text{xy}}+\lambda_{\text{xy}}.$$

The notation *v*^′^ and *w*^′^ is used to denote that these variables are not yet regularly distributed due to the small magnetostriction terms λ_xx_ and λ_xy_, which depend on *B*_x_, making *v*^′^ and *w*^′^ dependent on *u*. However, for each given value of *u*, we can easily interpolate new values $H_{\text{x}}^{\prime }$, $\sigma_{\text{xx}}^{\prime }$, $\sigma_{\text{xy}}^{\prime },\;\lambda_{\text{xx}}^{\prime }$ and $\lambda_{\mathrm{xy}}^{\prime }$ for the field, stress and magnetostriction components using simply one-dimensional interpolation so that *v* and *w* become regularly distributed:

2.16 $$u=B_{\text{x}},\;v=\frac{1+\nu }{E}\sigma_{xx}^{\prime }+\frac{3}{2}\lambda_{\text{xx}}^{\prime }\;\text{and}\;w=\frac{1+\nu }{E}\sigma_{\text{xy}}^{\prime }+\lambda_{\text{xy}}^{\prime }.$$

With these variables, we finally obtain

2.17 $$\frac{\partial \phi }{\partial u}=H_{\text{x}}^{\prime },\;\frac{\partial \phi }{\partial v}=-\frac{E}{1+\nu }\lambda_{\text{xx}}^{\prime }=-\tau_{\text{xx}}\;\text{and}\;\frac{\partial \phi }{\partial w}=-\frac{2E}{1+\nu }\lambda_{\text{xy}}^{\prime }=-2\tau_{\text{xy}},$$

where the last two terms are magnetostrictive parts of the stress (see appendix B). For brevity, these are denoted by *τ*_xx_ and *τ*_xy_. Again, the factor 2 in the last term results from making the symmetry assumption before differentiation. The splines can then be fitted similarly to the previous section by solving the overdetermined problems

2.18 $$\begin{array}{rl} & \left\{ \begin{array}{l} {H^{\prime }}_{\text{x},ij}=D_{io}^{\text{u}}A_{jm}^{\text{v}}c_{om}\\ \tau_{\text{xx}}=-A_{il}^{\text{u}}D_{jo}^{\text{v}}c_{lo} \end{array}\right.\\ \;\text{and}\; & \left\{ \begin{array}{l} {H^{\prime }}_{\text{x},ijk}=D_{io}^{\text{u}}A_{jm}^{\text{v}}A_{kn}^{\text{w}}c_{omn}\\ \tau_{\text{xx},ijk}=-A_{il}^{\text{u}}D_{jo}^{\text{v}}A_{kn}^{\text{w}}c_{lon}\\ \tau_{\text{xy},ijk}=-\frac{1}{2}A_{il}^{\text{u}}A_{jm}^{\text{v}}D_{ko}^{\text{w}}c_{lmo}. \end{array}\right. \end{array}$$

### (d) Implementation considerations

The fitting of the splines was implemented in Matlab^1^ (v.R2017b). For the given measurement grid *u*, *v* and *w* and the desired spline order, the B-spline knots were calculated with the Matlab function **optknt**. Given the grid, the order and the knots, the collocation matrices ***A***^u^, ***A***^v^ and ***A***^w^ in (2.5) were obtained with the function **spcol**. In order to construct the collocation matrices ***D***^u^, ***D***^v^ and ***D***^w^ in (2.6), **spcol** was first used to obtain collocation matrices for mapping the partial derivative B-spline coefficients into the measured partial derivative values. These were multiplied with matrices obtained by function **DerivBKnotDeriv** (found in [41]), which map the B-spline coefficients into partial derivative B-spline coefficients.

Different scaling factors should be chosen for different equations in the systems (2.6) and (2.18) in order to equalize their weighting and significance during the least-squares solution. For example, if in the bivariate case in (2.6), *B*_x,*ij*_ are expressed in teslas and λ_xx_,*_ij_* in m m^−1^, the magnetostriction has negligible effect on the least-squares solution, since its numerical values are in the range of 10^−6^. In our implementation, instead of solving (11), we divided the equations by the maximum corresponding measurement values:

2.19 $$\begin{array}{rl} & \left\{ \begin{array}{l} \frac{B_{\text{x},ij}}{\underset{ij}{\max}(|B_{\text{x, }ij}|)}=\frac{D_{io}^{\text{u}}A_{jm}^{\text{v}}}{\underset{ij}{\max}(|B_{\text{x, }ij}|)}c_{om}\\ \frac{\lambda_{\text{xx, }ij}}{\underset{ij}{\max}(|\lambda_{\text{xx, }ij}|)}=\frac{A_{il}^{\text{u}}D_{jo}^{\text{v}}}{\underset{ij}{\max}(|\lambda_{\text{xx, }ij}|)}c_{lo} \end{array}\right.\\ \;\text{and}\; & \left\{ \begin{array}{l} \frac{B_{\text{x},ijk}}{\underset{ijk}{\max}(|B_{\text{x},ijk}|)}=\frac{D_{io}^{\text{u}}A_{jm}^{\text{v}}A_{kn}^{\text{w}}}{\underset{ijk}{\max}(|B_{\text{x},ijk}|)}c_{omn}\\ \frac{\lambda_{\text{xx},ijk}}{\underset{ijk}{\max}(|\lambda_{\text{xx},ijk}|)}=\frac{A_{il}^{\text{u}}D_{jo}^{\text{v}}A_{kn}^{\text{w}}}{\underset{ijk}{\max}(|\lambda_{\text{xx},ijk}|)}c_{lon}\\ \frac{2\lambda_{\text{xy},ijk}}{\underset{ijk}{\max}(|\lambda_{\text{xy},ijk}|)}=\frac{A_{il}^{\text{u}}A_{jm}^{\text{v}}D_{ko}^{\text{w}}}{\underset{ijk}{\max}(|\lambda_{\text{xy},ijk}|)}c_{lmo} \end{array}\right., \end{array}$$

and thus equalized the weighting for the flux density and magnetostriction.

The full equation system can be written as ***Ac*** = ***b***, where the vector ***c*** includes the spline coefficients, ***A*** is the system matrix and vector ***b*** includes the scaled measurements. The sizes of ***A***, ***c*** and ***b*** are 2*N*_u_*N*_v_ × *M*_u_*M*_v_, *M*_u_*M*_v_ and 2*N*_u_*N*_v_ in the bivariate case and 3*N*_u_*N*_v_*N*_w_ × *M*_u_*M*_v_*M*_w_, *M*_u_*M*_v_*M*_w_ and 3*N*_u_*N*_v_*N*_w_ in the trivariate case. However, since only the partial derivatives of the energy are measured, this system does not have a unique solution. A constant could be added to the energy without changing the partial derivatives. Thus, one of the spline coefficients has to be fixed to a constant to obtain a unique solution. In our implementation, this is done by writing the least-squares systems only for *c_lm_* with *l* + *m* > 2 and *c_lmn_* with *l* + *m* *+* *n* > 3 in the bi- and trivariate cases, respectively, fixing manually *c*_11_ = 0 and *c*_111_ = 0. Thus the system matrix ***A*** and the unknown vector ***c*** are changed to ***A***′ and ***c***′ by removing the first column and the first row, respectively. The least-squares solution can then be obtained as

2.20 $$c^{\prime }={\left({A^{\prime }}^{\text{T}}A^{\prime }\right)}^{-1}{A^{\prime }}^{\text{T}}b\;\text{and}\;c=\left[\begin{array}{c} 0\\ c^{\prime } \end{array}\right].$$

The ranks and condition numbers of the system matrices before (*r*(***A***) and *κ*(***A***)) and after (*r*(***A***′) and *κ*(***A***′)) removing the columns corresponding to *c*_11_ and *c*_111_ will be compared in the results section to give insight on the uniqueness of the solution.

### (e) Simplified multiscale model

It is important to verify if the thermodynamic constitutive law can be identified from measurements under *H*, $\sigma_{\mathrm{xx}}$ and (possibly) $\sigma_{\mathrm{xy}}$ in such a way that it can reasonably reproduce the magneto-elastic behaviour under arbitrary multiaxial loadings. Such a verification would require an extensive amount of measurements in complex loading conditions in three dimensions, which are not currently possible. Thus, the simplified three-dimensional multiscale material model developed in [17] is used as a reference. The SMS model is applied to produce simulation data for both identification and validation of the thermodynamic constitutive law.

The SMS model is based on expressing a local potential energy for a domain *α* oriented along a given direction ***u****_α_* as a function of the field strength ***H*** and stress ***σ*** as

2.21 $$W_{\alpha }=-\mu_{0}H\cdot M_{\alpha }-\sigma :\lambda_{\alpha },$$

where

2.22 $$M_{\alpha }=M_{\text{s}}u_{\alpha }\;\text{and}\;\lambda_{\alpha }=\frac{3}{2}\lambda_{\text{s}}\left(u_{\alpha }⊗u_{\alpha }-\frac{1}{3}I\right),$$

are the magnetization and magnetostriction of the given domain, determined by the saturation values *M*_s_ and λ_s_, respectively. The volume fraction of domains is expressed as

2.23 $$f_{\alpha }=\frac{\exp(-A_{\text{s}}W_{\alpha })}{\int_{\alpha }\exp(-A_{\text{s}}W_{\alpha })}\;\text{with }A_{\text{s}}=\frac{3\chi_{0}}{\mu_{0}M_{\text{s}}^{\text{2}}},$$

where *χ*_0_ is the initial magnetic susceptibility of the material. The magnetization vector and magnetostriction tensor are then obtained by weighting the domain magnetizations and magnetostrictions by the volume fraction and integrating over all possible orientations as

2.24 $$M=\int_{\alpha }f_{\alpha }M_{\alpha }\;\text{and}\;\lambda =\int_{\alpha }f_{\alpha }\lambda_{\alpha }.$$

In our implementation, the integrations over two direction angles defining a unit spherical shell are carried out numerically using adaptive two-dimensional quadratures. The magnetic flux density is obtained as ***B*** = *μ*_0_(***H*** + ***M***).

## 3. Application and results

### (a) Fitting of the splines

Reference identification data for *N*_u_ = 40 and *N*_v_ = *N*_w_ = 11 values of *B*_x_(*H*_x_, $\sigma_{\mathrm{xx}}$) and λ_xx_(*H*_x_, $\sigma_{\mathrm{xx}}$) as well as *B*_x_(*H*_x_, $\sigma_{\mathrm{xx}}$, $\sigma_{\mathrm{xy}}$), λ_xx_(*H*_x_, $\sigma_{\mathrm{xx}}$, $\sigma_{\mathrm{xy}}$) and λ_xy_(*H*_x_, $\sigma_{\mathrm{xx}}$, $\sigma_{\mathrm{xy}}$) was produced with the SMS model described in §2e. The parameters of the SMS model were *M*_s_ = 1.45 MA m^−1^, λ_s_ = 6.67 µm m^−1^ and *χ*_0_ = 1585, representing a typical FeSi electrical steel. The bi- and trivariate splines *ϕ*^(2)^ and *ϕ*^(3)^ in terms of ***H*** and ***σ*** were fitted against the data as described in §2b, using an order of 3 for the B-spline and as many spline coefficients as measurement points: *M*_u_ = *N*_u_, *M*_v_ = *N*_v_ and *M*_w_ = *N*_w_. The fitting errors were calculated separately for *B*_x_, λ_xx_ and λ_xy_ as

3.1 $$r_{\text{fit}}=\frac{||x-x_{\text{fit}}||}{||x||},$$

where vector ***x*** contains the multiscale model results in the *N*_u_ × *N*_v_ = 440 or *N*_u_ × *N*_v_ × *N*_w_ = 4840 measurement points, and vector ***x***_fit_ contains the corresponding results obtained by differentiating the fitted spline.

**Figure 4.**
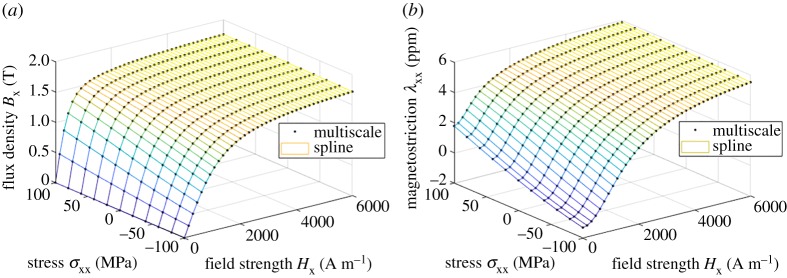
Fitting of the energy density *ϕ*^(2)^(***H***, ***σ***) as a bivariate spline to the results of the multiscale model: (*a*) flux density *B*_x_ and (*b*) magnetostriction λ_xx_. (Online version in colour.)

**Figure 5.**
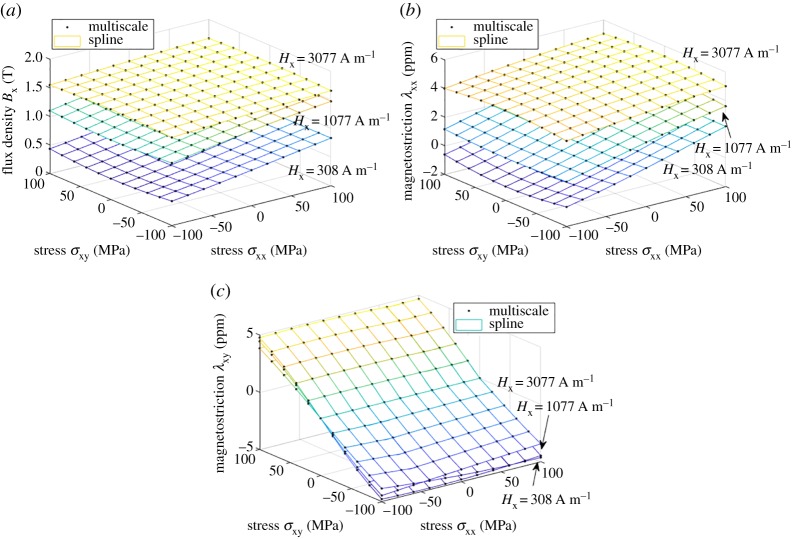
Fitting of the energy density *ϕ*^(3)^(***H***, ***σ***) as a trivariate spline to the results of the multiscale model: (*a*) flux density *B*_x_, (*b*) magnetostriction λ_xx_ and (*c*) magnetostriction λ_xy_. The results are shown at three different field strength values *H*_x_. (Online version in colour.)

Figure 4 shows the fitting results for the bivariate spline *ϕ*^(2)^(***H***, ***σ***). In this case, the fitting errors *r*_fit_ for *B*_x_ and λ_xx_ were 0.0037% and 0.0059%, respectively, which means that a single energy function can represent both the flux density and magnetostriction consistently to the SMS model under uniaxial loading. The ranks and condition numbers of the system matrix are *r*(***A***) = 339 and *κ*(***A***) = 8.3 × 10^17^ before and *r*(***A***′) = 339 and *κ*(***A***′) = 3.1×10^4^ after removing the column corresponding to coefficient *c*_11_ which is fixed manually to zero. The unchanged rank and the large drop in the condition number show that the system becomes well-conditioned after removing one column, making the solution unique. Figure 5 shows the fitting results in the trivariate case *ϕ*^(3)^(***H***, ***σ***) for three different values of the field strength *H*_x_. In this case, the errors for *B*_x_, λ_xx_ and λ_xy_ were 0.0041%, 0.0059% and 0.0017%, respectively. The ranks and condition numbers are *r*(***A***) = 4839 and *κ*(***A***) = 6.1×10^17^ and *r*(***A***′) = 4839 and *κ*(***A***′) = 2.7 × 10^5^, again showing the uniqueness of the solution after fixing *c*_111_. Noteworthy is that the error does not increase from the bivariate case, which means that also the behaviour under shear stress is similarly predicted by the thermodynamic and SMS models. The agreement between the two models is surprisingly good.

From figure 4*b*, it can be seen that the SMS model produces magnetostriction under stress even when the magnetic field is zero. This behaviour is explained as the so-called Δ*E* effect [42], which can be observed in ferromagnetic materials, and is often described as a change in the Young modulus *E* under stress. The spline-based thermodynamic model is able to reproduce this behaviour contrary to the analytical energy density functions (1.5) we have used earlier.

**Figure 6.**
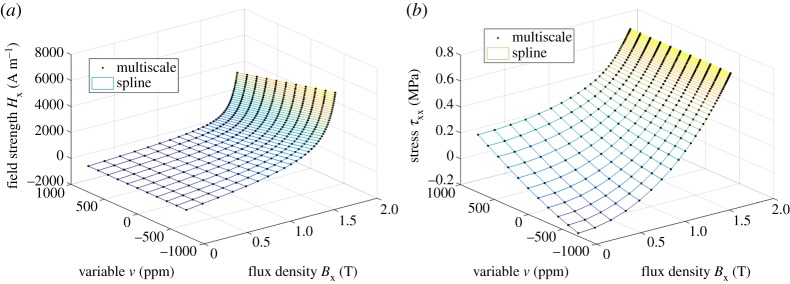
Fitting of the energy density *ϕ*^(2)^(***B***, ***ε***) as a bivariate spline to the results of the multiscale model: (*a*) field strength *H*_x_ and (*b*) stress *τ*_xx_ from (2.17). (Online version in colour.)

**Figure 7.**
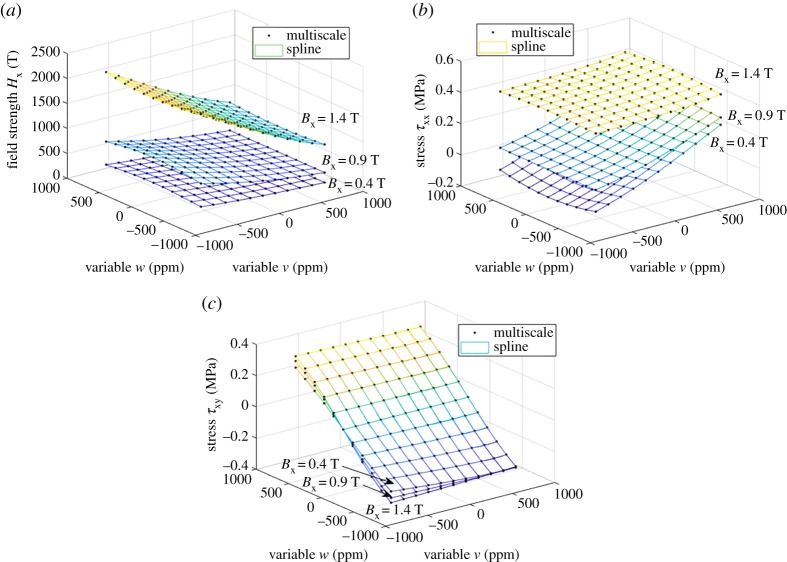
Fitting of the energy density *ϕ*^(3)^(***B***, ***ε***) as a trivariate spline to the results of the multiscale model: (*a*) field strength *H*_x_, (*b*) stress *τ*_xx_ and (*c*) stress *τ*_xy_ from (2.17). The results are shown at three different flux density values *B*_x_. (Online version in colour.)

The same SMS results were next interpolated into the form *H*_x_(*B*_x_, $\sigma_{\mathrm{xx}}$, $\sigma_{\mathrm{xy}}$), λ_xx_(*B*_x_, $\sigma_{\mathrm{xx}}$, $\sigma_{\mathrm{xy}}$) and λ_xy_(*B*_x_, $\sigma_{\mathrm{xx}}$, $\sigma_{\mathrm{xy}}$), and the bi- and trivariate splines were fitted in terms of ***B*** and ***ε***, as described in §2c, again with an order of 3. The values used for the Young modulus and the Poisson ratio were *E* = 183 GPa and *ν* = 0.34. Figure 6 shows the fitting results for the bivariate spline *ϕ*^(2)^(***B***, ***ε***). In this case, the fitting errors for *H*_x_ and *τ*_xx_ were 0.019% and 0.016%, respectively. Figure 7 shows the fitting results in the trivariate case *ϕ*^(3)^(***B***, ***ε***) for three different values of the flux density *B*_x_. In this case, the errors for *H*_x_, *τ*_xx_ and *τ*_xy_ were 0.020%, 0.017% and 0.009%, respectively. The ranks and condition numbers behave similarly to the previous case, ensuring the uniqueness of the solutions. The errors grow slightly from the ***H***- and ***σ***-based model, mainly due to the required numerical interpolations. Nevertheless, the spline-based approach is clearly able to cope with different combinations of input variables.

### (b) Sensitivity analysis

It is important to study the robustness of the fitting procedure against measurement errors and noise, which are inevitable in real experiments. When measurements of magnetization and magnetostriction curves are obtained, the data are usually smoothed and denoised in a preprocessing stage before fitting constitutive models. Such preprocessing is likely to alter the relationship between the magnetization and magnetostriction curves in such a way that they cannot be exactly described by a single thermodynamic potential. To study the sensitivity of the fitting procedure against such errors, artificial error is introduced to the SMS model results by changing the output flux density and magnetostriction as

**Figure 8.**
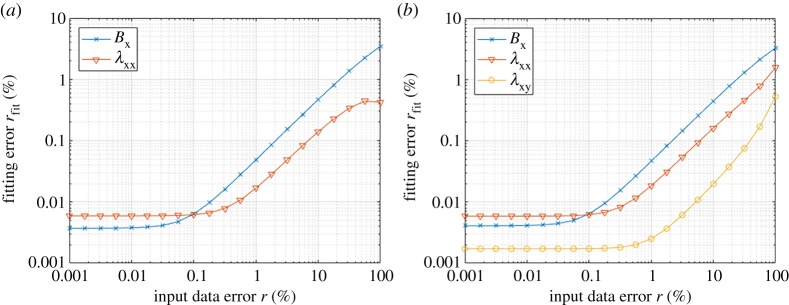
Comparison of the fitting errors when the input data are modified by an error of *r* according to (3.2) in (*a*) the bivariate *ϕ*^(2)^(***H***, ***σ***) and (*b*) the trivariate case *ϕ*^(3)^(***H***, ***σ***). (Online version in colour.)

3.2 $$B_{\text{x}}\to \left(1+\frac{r}{2}\right)B_{\text{x}}\;\text{and}\;\lambda_{\text{xx}}\to \left(1-\frac{r}{2}\right)\lambda_{\text{xx}},$$

where the error *r* varies between 0.001% and 100%. λ_xy_ is kept unchanged in the trivariate case. The fitting of the bivariate and trivariate splines *ϕ*^(2)^ and *ϕ*^(3)^ in terms of ***H*** and ***σ*** is repeated similarly to the previous section, and the fitting errors *r*_fit_ from (3.1) are studied as a function of *r*. The results are shown in figure 8. It is seen that the fitting errors *r*_fit_ increase slowly with respect to *r* and remain below 10% for each case when *r* < 100%. The fitting procedure is thus robust and able to find a reasonable thermodynamic potential even for the case where the input data do not exactly fulfil the thermodynamic principles.

### (c) Comparison of models: field in principal stress direction

After fitting the splines, the behaviour of the proposed spline-based thermodynamic model under multiaxial stresses is compared to that of the SMS model. Both the SMS model and the thermodynamic model in terms of ***H*** and ***σ*** are simulated under a magnetic field of ***H*** = [*H*_x_ 0 0]^T^ with *H*_x_ = 1077 A m^−1^ and the following multiaxial stresses, expressed in the Voigt notation:

$$\begin{array}{cc} \text{uniaxial}: & \sigma =[\sigma 0\;0\;0\;0\;0],\\ \text{equibiaxial:} & \sigma =[\sigma \sigma 0\;0\;0\;0],\\ \text{hydrostatic:} & \sigma =[\sigma \sigma \sigma 0\;0\;0],\\ \text{pure}\text{shear:} & \;\;\sigma =[\sigma \;-\sigma 0\;0\;0\;0]. \end{array}$$

These stresses have principal axes parallel to the *x*-, *y*- and *z*-axes, and thus the magnetic flux densities simulated with the two models become parallel to the field strength, which allows comparing the models in terms of the relative permeability.

**Figure 9.**
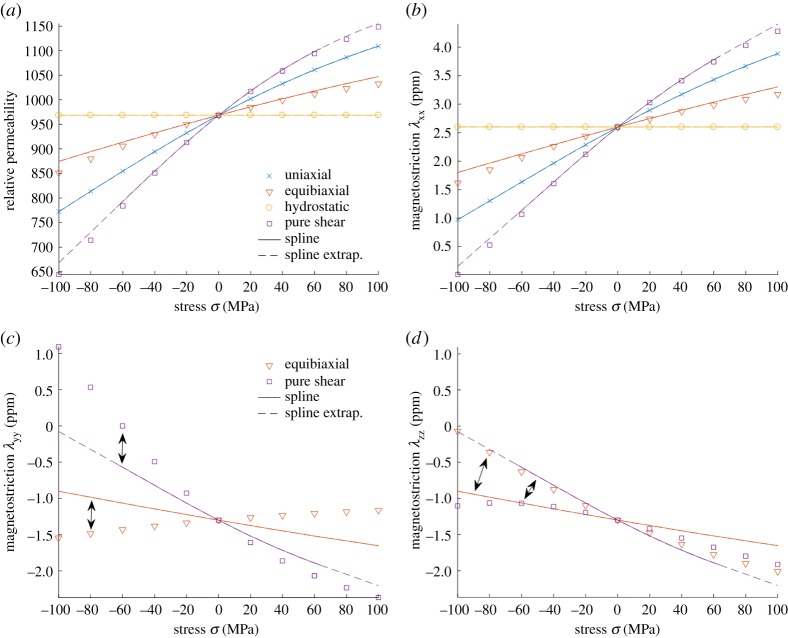
Comparison of (*a*) the relative permeabilities and magnetostrictions, (*b*) λ_xx_, (*c*) λ_yy_ and (*d*) λ_zz_ simulated with the simplified multiscale model (markers) and the bivariate spline-based thermodynamic model under multiaxial stresses. The dashed line shows the region in which extrapolation is used. The results with the trivariate spline are almost equal to the bivariate ones. In (*c*,*d*), the arrows denote which markers correspond to which curves. (Online version in colour.)

Figure 9*a*,*b* compares the relative permeability and magnetostriction λ_xx_ (component parallel to the field) simulated with the SMS model and the bivariate spline. The results with the trivariate spline are almost equal to the bivariate ones and thus not separately shown. Both models behave very similarly, and the extrapolation of the spline appears to work sufficiently. However, when the magnetostrictions λ_yy_ and λ_zz_ (components perpendicular to the field) are compared in figure 9*c*,*d* in the cases of equibiaxial and pure shear loadings, rather significant differences are observed. The spline-based thermodynamic model produces exactly the same magnetostrictions in the *yy*- and *zz*-directions, despite the fact that the stress affects only in the *xy*-plane. This is a property of the invariant model and directly results from the fact that under a uniaxial field ***H*** = [*H*_x_ 0 0]^T^ and an arbitrary stress tensor ***σ***, the invariants *I*_5_ and *I*_6_ become

3.3 $$I_{5}=\frac{1}{3}(2\sigma_{\text{xx}}-\sigma_{\text{yy}}-\sigma_{\text{zz}})H_{\text{x}}^{2}\;\text{and}\;I_{6}=\left[\frac{1}{9}{\left(2\sigma_{\text{xx}}-\sigma_{\text{yy}}-\sigma_{\text{zz}}\right)}^{2}+\sigma_{\text{xy}}\sigma_{\text{yx}}+\sigma_{\text{xz}}\sigma_{\text{zx}}\right]H_{\text{x}}^{2},$$

leading to

3.4 $$\frac{\partial I_{5}}{\partial \sigma_{\text{yy}}}=\frac{\partial I_{5}}{\partial \sigma_{\text{zz}}}\;\text{and}\;\frac{\partial I_{6}}{\partial \sigma_{\text{yy}}}=\frac{\partial I_{6}}{\partial \sigma_{\text{zz}}},$$

and thus λ_yy_ = λ_zz_. The effect of the stress is thus transversely isotropic in the plane perpendicular to the field. This is not the case with the SMS model, in which the effect of the stress on the magnetostriction is anisotropic even in the plane perpendicular to the field. Three-dimensional measurements under multiaxial loadings would be needed in order to validate the model predictions. Both models produce volume-preserving magnetostriction, such that λ_xx_ + λ_yy_ + λ_zz_ = 0.

### (d) Comparison of models: shear stress with respect to field

In the previous section, the field strength was oriented along the principal axes of the stress tensor. This means that the flux density and field strength vectors remain parallel. To compare the models under conditions in which the stress-induced anisotropy rotates the flux density with respect to the field strength, the SMS model and the thermodynamic models were simulated under a field strength of ***H*** = [*H*_x_ 0 0]^T^ with *H*_x_ = 1077 A m^−1^ and a shear stress tensor

3.5 $$\sigma (\theta )=T(\theta )\left[\begin{array}{ccc} 0 & \sigma_{\text{xy}} & 0\\ \sigma_{\text{xy}} & 0 & 0\\ 0 & 0 & 0 \end{array}\right]T{\left(\theta \right)}^{\text{T}}\;\text{with }T(\theta )=\left[\begin{array}{ccc} \cos\theta & -\sin\theta & 0\\ \sin\theta & \cos\theta & 0\\ 0 & 0 & 1 \end{array}\right],$$

which can be rotated in the *xy*-plane by varying angle *θ*. The magnitude of the stress is $\sigma_{\mathrm{xy}}$ = 50 MPa, and *θ* is varied from 0 to 180°.

**Figure 10.**
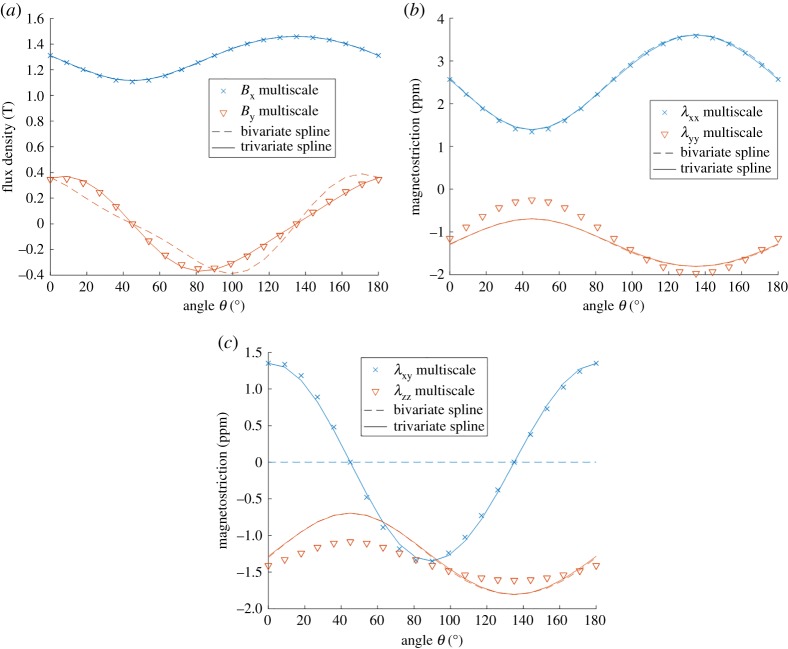
Comparison of (*a*) flux density components *B*_x_ and *B*_y_, (*b*) magnetostrictions λ_xx_ and λ_yy_, and (*c*) magnetostrictions λ_xy_ and λ_zz_ simulated with the simplified multiscale model and the bi- and trivariate spline-based thermodynamic models under pure shear stress oriented in different angles in the *xy*-plane according to (3.5). (Online version in colour.)

Figure 10*a* compares the magnetic flux density components *B*_x_ and *B*_y_ produced by the SMS and thermodynamic models at different angles *θ*. As expected, the bivariate spline model fails to accurately present the behaviour under shear stress, since the shear is not accounted for during the identification process. On the contrary, the flux density produced by the trivariate model agrees well with the SMS model. The magnetostrictions λ_xx_ and λ_yy_ are compared in figure 10*b*. Similarly to the results of figure 9*c*, λ_yy_ differs clearly between the two models, and the bi- and trivariate models agree well. Finally, the magnetostrictions λ_xy_ and λ_zz_ are compared in figure 10*c*. The bivariate model produces zero λ_xy_, while the trivariate model agrees well with the SMS model. λ_zz_ differs again similarly to figure 9*d*.

### (e) Fitting to measurement results

**Figure 11.**
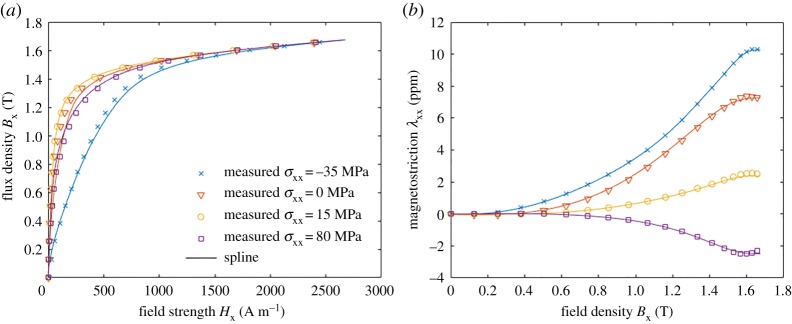
Fitting of the energy density *ϕ*^(2)^(***B***, ***ε***) as a bivariate spline to the measurement results shown in figure 2: (*a*) field strength *H*_x_ and (*b*) magnetostriction λ_xx_. (Online version in colour.)

The proposed thermodynamic approach was also tested against the uniaxial measurements discussed in §2a and figure 2. Following the idea described in §2c, the model was fitted in terms of ***B*** and ***ε*** as a bivariate spline *ϕ*^(3)^(***B***, ***ε***). *N*_u_ = *M*_u_ = 100 and *N*_v_ = *M*_v_ = 17 values were used for *B* and variable *v*, and the spline coefficients, respectively. The results are shown in figure 11. Both the magnetization curves and the magnetostriction are satisfactorily fitted, the fitting errors for *B*_x_ and *τ*_xx_ being 2.5% and 1.9%, respectively. However, when comparing to the analytical model in figure 3, the fitting is significantly better. Since the spline-based model does not make any assumptions on the shape of the energy density, the only factor affecting the quality of the fit is the correctness of the assumption, that a single thermodynamic potential is able to describe the magnetization and magnetostriction simultaneously.

## 4. Discussion and conclusion

An isotropic spline-based thermodynamic approach was presented for modelling coupled magneto-mechanical behaviour in ferromagnetic materials. The model is based on defining an energy-density functional, which depends on the invariant basis defined by the input vector and tensor, but assumptions on the exact form of the energy density are not made. The model is thus flexible and allows choosing freely the state variables, as a function of which the energy density is expressed. Flux density and strain are convenient choices, when the model is applied in finite-element analysis using formulations with magnetic vector potential and mechanical displacement. Magnetic field can be used in magnetic scalar potential formulations or if the model is to be coupled to a Jiles-Atherton hysteresis model, as in [36]. If the stress is known during the measurements, using it as a state variable simplifies the model derivations and the identification. The proposed identification procedure is not limited to magneto-elastic behaviour but can most likely be used also for other coupled problems, in which the constitutive laws are based on defining a free energy density and suitable measurements are available.

Comparison to the SMS model in the bi- and trivariate cases showed that both the multiscale model and the thermodynamic approach produce very similar magnetization curves. This has not been observed earlier when using analytical energy-density expressions, since these have prevented accurately fitting the thermodynamic model. The excellent agreement in the magnetization curves and magnetostrictions parallel to the magnetic field is rather surprising, considering that the two models are based on different modelling considerations. The observed differences in the magnetostrictions perpendicular to the field should be further studied and validated by measurements.

The main advantage of the multiscale models is that they rely on few intrinsic parameters, which have clear physical meaning and which are rather easy to obtain from measurements. However, deriving the models with input variables other than the magnetic field and stress is difficult, and it is thus complicated to implement the models in numerical tools. Since the proposed thermodynamic approach can be rather easily fitted against the multiscale model results, it could be used as a tool for simplifying the numerical implementation while accurately preserving the constitutive behaviour obtained from the multiscale model.

When fitting the spline-based model against measurement results, measurement error and noise obviously affect the quality of the fit. Experiments have shown that downsampling the measurement results and increasing the order of the B-spline make the fitted splines less prone to oscillations. In addition, the fitting can be improved by increasing the weight of, for example, the magnetization curve with respect to the magnetostriction curve before the least-squares solution.

If an isotropic material can be assumed, the three invariants *I*_4_, *I*_5_ and *I*_6_ are enough to describe the behaviour under arbitrary three-dimensional loadings based on the measurements in the regular grid described by the auxiliary variables *u*, *v*, *w*. Orthotropic anisotropy may be accounted for by considering two additional direction vectors describing two perpendicular symmetry planes and extending the scalar integrity basis of the free energy density to account for these vectors. This will result in 19 scalar invariants. In theory, a similar spline-based identification approach would be possible also in the anisotropic case, but a huge amount of measurement data would be required to identify the splines. The model could be further extended to plastic deformation by considering the equivalent plastic strain as an additional invariant, but identification of such a model would also require new measurements. To simplify the identification process, these measurements could also be replaced by multiscale models, which can account for anisotropy [43] and plastic deformation [44].

In this work, the Maxwell stress was neglected from both the thermodynamic approach and the SMS approach. The Maxwell stress could be considered in the momentum balance equations when solving coupled magneto-mechanical field problems. The Maxwell stress is obviously included in the measurements against which the thermodynamic model was fitted. However, considering the maximum values of *B*_x_ and *H*_x_ in figure 2*a*, we can estimate that the magnitude of the Maxwell stress is around 1.67 T×680 A m^−1^ ≈ 4490 Pa. This value is about 4 magnitudes lower than the applied loadings and can thus be neglected.

## Footnotes

1 The codes can be found at https://github.com/prasilo/magnetostriction-spline/tree/v2.0.

## Data accessibility

Matlab codes used for producing the results can be found at https://github.com/prasilo/magnetostriction-spline/tree/v2.0.

## Appendix A

The Lamé parameters are obtained from Young's modulus *E* and Poisson's ratio *ν* as

A 1 $$\lambda =\frac{E\nu }{\left(1+\nu )(1-2\nu \right)}\;\text{and}\;\mu =\frac{E}{2(1+\nu )}.$$

λ is not to be mixed with the magnetostriction tensor and its components, which are denoted **λ**, λ_xx_, λ_yy_, λ_zz_ and λ_xy_.

## Appendix B

The magnetostricive parts of the stress appearing in (2.17) are derived here. Comparing (2.12) and (2.13) to (2.16), we can see that

B 1 $$v=\frac{3}{2}e_{\text{xx}}\;\text{and}\;w=\varepsilon_{\text{xy}}.$$

Let us divide the total energy density into mechanical and magneto-mechanical parts:

B 2 $$\psi (B,\varepsilon )=\phi_{\text{mech}}(\varepsilon )+\phi (B,e)\;\text{with }\phi_{\text{mech}}=\frac{1}{2}\lambda I_{1}^{2}+\mu I_{2}.$$

Differentiating this total energy density with respect to the xx component of the total strain gives the xx component of the stress

B 3 $$\sigma_{\text{xx}}=\frac{\partial \psi }{\partial \varepsilon_{\text{xx}}}=\frac{\partial \phi_{\text{mech}}}{\partial \varepsilon_{\text{xx}}}+\frac{\partial \phi }{\partial e_{\text{xx}}}\frac{\partial e_{\text{xx}}}{\partial \varepsilon_{\text{xx}}}=\lambda I_{1}+2\mu \varepsilon_{\text{xx}}+\frac{2}{3}\frac{\partial \phi }{\partial e_{\text{xx}}}=\lambda I_{1}+2\mu \varepsilon_{\text{xx}}+\frac{\partial \phi }{\partial v},$$

where (B 1) was used. By rearranging, we get

B 4 $$\frac{\partial \phi }{\partial v}=\sigma_{\text{xx}}-\lambda I_{1}-2\mu \varepsilon_{\text{xx}}.$$

Substituting here λ and *μ* from (A 1), the strain components from (2.11), *I*_1_ from (1.3) as well as the isochoric magnetostriction from (2.10) yields

B 5 $$\frac{\partial \phi }{\partial v}=-\frac{E}{\left(1+\nu \right)}\lambda_{\text{xx}},$$

which is the contribution of the magnetostriction to the stress. With positive magnetostriction, compressive stress is created.

With a similar reasoning but taking into account that each shear component contributes twice to the energy due to symmetry of the stress and strain tensors, we get

B 6 $$\frac{1}{2}\frac{\partial \phi }{\partial w}=\sigma_{\text{xy}}-2\mu \varepsilon_{\text{xy}}.$$

Substituting here *μ* from (A 1) and the shear strain from (2.13) yields

B 7 $$\frac{\partial \phi }{\partial w}=-\frac{2E}{\left(1+\nu \right)}\lambda_{\text{xy}}.$$
